# Multi-omics technology reveals the changes in gut microbiota to stimulate aromatic amino acid metabolism in children with allergic rhinitis and constipation

**DOI:** 10.3389/falgy.2025.1562832

**Published:** 2025-05-09

**Authors:** Chunyan Wang, Haiying Liu, Xiaoli Li, Wei Kong, Hui Wu, Congfu Huang

**Affiliations:** ^1^ Department of Pediatrics, The Fourth People's Hospital of Shenzhen, Shenzhen, China; ^2^ Department of Pediatrics, Affiliated Shenzhen Maternity and Child Healthcare Hospital, Southern Medical University, Shenzhen, China; ^3^ Department of Pediatrics, Longgang District Maternity & Child Healthcare Hospital of Shenzhen City (Longgang Maternity and Child Institute of Shantou University Medical College), Shenzhen, China; ^4^ Child Healthcare Department, Panyu Maternal and Child Care Service Centre, Guangzhou, China

**Keywords:** allergic rhinitis, constipation, gut microbiota, aromatic amino acids, high-throughput absolute quantification, metabolomics

## Abstract

**Background:**

Comorbid allergic rhinitis and constipation (ARFC) in children are associated with gut microbiota (GM) dysbiosis and metabolic perturbations; however, the underlying mechanistic interplay remains unclear.

**Objective:**

This multi-omics study aimed to characterize GM and fecal metabolomic signatures in preschool ARFC children and elucidate microbial–metabolite interactions driving dual symptomatology.

**Methods:**

Fecal samples from 16 ARFC and 15 healthy control (HC) children underwent high-throughput absolute quantification 16S rRNA sequencing and untargeted metabolomics. Differential taxa and metabolites were identified via LEfSe and OPLS-DA (VIP > 1, false discovery rate (FDR) *q* < 0.05). Microbial–metabolite networks were reconstructed using genome-scale metabolic modeling and KEGG pathway analysis.

**Results:**

The ARFC group exhibited distinct β-diversity (*P* = 0.031), marked by elevated *Hungatella*, *Tyzzerella*, and *Bifidobacterium longum* (*P* < 0.05). Metabolomics revealed upregulated aromatic amino acids (AAAs), neurotransmitters, and bile acids (FDR *q* < 0.05), with enrichment in tryptophan/tyrosine pathways (*P* < 0.01). Bioinformatic modeling linked *Hungatella* to tryptophan hydroxylase (EC:1.14.16.4), driving serotonin synthesis, and *Tyzzerella* to indoleamine 2,3-dioxygenase (EC:1.13.11.52), promoting kynurenine production. *Bifidobacterium longum* correlated with phenylalanine hydroxylase (EC:1.14.16.1), enhancing phenylalanine derivatives. A combined GM–metabolite diagnostic model demonstrated robust accuracy (AUC = 0.8).

**Conclusion:**

GM dysbiosis in ARFC children activates AAA metabolism, generating neuroactive and pro-inflammatory metabolites that may exacerbate allergic and gastrointestinal symptoms. These findings highlight microbial–metabolite axes as therapeutic targets. Study limitations include cohort size and lack of disease-specific controls, necessitating validation in expanded cohorts.

## 1 Background

Allergic rhinitis (AR) is a chronic non-infectious inflammatory condition of the nasal mucosa, primarily mediated by allergen-specific IgE in sensitized individuals following allergen exposure. Non-IgE mechanisms and neuroimmune dysregulation also contribute to its pathogenesis. Environmental factors and gut microbiota (GM) play significant roles in AR development (1), and genetic susceptibility further influences its occurrence (2). Typical symptoms of AR include paroxysmal sneezing, watery nasal discharge, nasal itching, and nasal congestion, often accompanied by ocular symptoms such as itching, tearing, redness, and burning. AR is particularly prevalent in individuals with pollen allergies, affecting 10%–20% of the global population and posing a significant public health challenge. The condition is associated with various comorbidities, including bronchial asthma and upper airway cough syndrome, and significantly impacts patients’ quality of life through sleep disturbances, emotional stress, and increased economic burden (2–5). Notably, children with AR are prone to constipation (5), and constipation itself may elevate the risk of AR (6, 7).

**Table 1:** Baseline characteristics of the ARFC group and HC group.

Characteristic	ARFC group (*n* = 16)	HC group (*n* = 15)	*p*-value
Age (years)	4.5 ± 1.2	4.3 ± 1.1	0.67
Gender (male/female)	9/7	8/7	0.89
Dietary fiber intake (g/day)	12.3 ± 2.5	13.1 ± 3.0	0.42
Antibiotic use (past 3 months)	0/16	0/15	–

GM dysbiosis is a recognized feature in AR patients and has emerged as a novel therapeutic target (8). Research by De Filippis et al. (9) demonstrated specific microbial signatures in AR children, such as an increased abundance of *Ruminococcus gnavus* and *Faecalibacterium prausnitzii* and reduced levels of *Bifidobacterium longum* and *Bacteroides dorei*, which are linked to heightened pro-inflammatory potential of GM. Additionally, reduced GM diversity has been associated with elevated serum IgE levels (10). Temporal variations in GM composition between disease onset and remission phases suggest that microbial shifts may influence AR pathogenesis (11). In AR children with functional gastrointestinal disorders, decreased levels of *Bifidobacterium*, *Lactobacillus*, gastrin, and motilin, alongside increased levels of *Enterobacter*, *Enterococcus*, somatostatin, and serotonin, have been observed (12). Interventions with glutamine and probiotics have shown promise in optimizing GM (12). Meta-analyses further support these findings, although the precise mechanisms underlying GM characteristics and AR pathogenesis remain elusive (13, 14).

Individuals with allergic rhinitis (AR) not only exhibit gut microbiota (GM) dysbiosis but also present metabolomic alterations. However, existing studies predominantly report on blood or urine metabolomics in AR populations, with limited data on fecal metabolomics (14, 15). For instance, Yuan et al. (14) identified 26 differential metabolites (e.g., prostaglandin D2, 20-hydroxy leukotriene B4, and linoleic acid) and disrupted 16 metabolic pathways (e.g., linoleic acid metabolism, arachidonic acid metabolism, and tryptophan metabolism) in adult AR patients through serum metabolomics. Blood metabolic pathway analysis highlighted enrichment in linoleic acid metabolism, arachidonic acid metabolism, and caffeine metabolism. A cross-ethnic Mendelian randomization study by Tu et al. (15) suggested that blood metabolites such as histidine, alanine, threonine, lysine, stearate, and 1-arachidonic acid glycerophosphate may be risk factors for AR, while kynurenine and 1-eicosadienoyl glycerophosphocholine could serve as protective factors. Urinary metabolic pathway analysis indicated associations between methylhistidine metabolism, glycine, serine metabolism, and AR, with the “α-linolenic acid and linoleic acid metabolism” pathway being the most significantly related to allergic diseases. Additionally, immunotherapy in AR patients with comorbidities has been shown to alter various amino acids and lipid metabolites, including arachidonic acid (16, 17).

In the context of functional constipation, Li et al. (18) reported altered levels of 79 metabolites, such as chenodeoxycholic acid and biliverdin, in the gut, with primary bile acid biosynthesis and porphyrin/chlorophyll metabolic profiles being the most enriched pathways. Luo et al. (19) observed reduced beneficial gut bacteria in loperamide-induced constipation in rats, accompanied by changes in fecal metabolic pathways related to carbohydrate, amino acid, energy, cofactor/vitamin, nucleotide, and lipid metabolism, as well as alterations in serum metabolites (including bile acids and steroids). Lactulose treatment for constipation in mice has been attributed to the reversal of GM function, promoting the production of intestinal metabolites such as bile acids, short-chain fatty acids, and tryptophan catabolites (20).

Our clinical observations in AR children revealed that approximately 20% of AR children also present with constipation, suggesting potential specific alterations in their GM. These GM-derived metabolites may influence the pathogenesis of both AR and constipation, a mechanism that remains unexplored. This study employs absolute GM quantification and non-targeted metabolomics to investigate differences in fecal bacterial diversity and metabolite levels in children with comorbid AR and constipation. Furthermore, we aim to elucidate the correlations between GM, metabolites, and clinical phenotypes. By constructing a representative discriminant model based on dominant genera and differential metabolites, we seek to identify marker microorganisms and metabolites associated with AR and constipation in children.

## 2 Materials and methods

### 2.1 Patient data and sample collection

This study was approved by the Longgang District Maternal and Child Health Hospital ethical review board (reference number KYXMLL-01-CZGC-14-2-1) and registered in the Chinese Clinical Trial Registry (ChiCTR2400085982). Conducted at the pediatric department of Longgang District Maternal and Child Health Hospital in Shenzhen, China, this study followed a comprehensive protocol with informed written consent obtained from the parents of all child participants. Enrollment, performed in the pediatric outpatient clinic from January to December 2024, included 16 preschool children (aged ≥3 to <7 years) diagnosed with allergic rhinitis (AR) and constipation (ARFC group) and 15 age-matched healthy controls (HC group). AR and constipation diagnoses were based on established criteria [(1, 21), respectively]. Participants were matched for age, sex, and dietary habits. The exclusion criteria encompassed severe gastrointestinal, hepatic, renal, or infectious diseases; antibiotic or probiotic use within the prior month; and developmental disorders such as autism spectrum disorder, to ensure minimizing confounding effects on GM. These criteria ensured no recent antibiotic or probiotic use, minimizing confounding effects on gut microbiota (GM). Fresh fecal samples were collected using sterile swabs, stored in airtight containers, and frozen at −80°C within 30 min.

Sample size determination was based on pilot study data (*n* = 6 per group), which demonstrated a Cohen's *d* effect size of 1.2 for β-diversity differences. Power analysis (*α* = 0.05, power = 0.8) indicated a minimum of 12 participants per group. Accounting for potential dropouts, 16 ARFC children and 15 HC children were enrolled. The HC group provided baseline GM and metabolite profiles for healthy children. While comparisons with AR-only or constipation-only groups could further clarify disease-specific interactions, such cohorts were not available in the current clinical setting and will be prioritized in future studies.

### 2.2 Absolute quantitative detection method for GM

#### 2.2.1 Sample DNA extraction

Fecal samples from the ARFC group and HC group were processed for DNA extraction using the FastPure Stool DNA Isolation Kit (MJYH, Shanghai, China) following the manufacturer's instructions. The integrity of the extracted microbial genomic DNA was assessed via 1% agarose gel electrophoresis, while DNA concentration and purity were measured using a NanoDrop 2000 spectrophotometer (Thermo Fisher Scientific, USA). To ensure accurate quantification, 12 synthetic DNA sequences (spike-in DNA) were added to the extracted DNA at four concentrations (10^3^, 10^4^, 10^5^, and 10^6^ copies). These sequences, which include a conserved region of the native 16S rRNA gene and a variable artificial segment, served as internal standards. Subsequently, a bacterial amplicon library was generated by amplifying the V3–V4 hypervariable regions of the bacterial 16S rRNA gene.

#### 2.2.2 PCR amplification and sequencing library construction

DNA samples from both groups were used as templates for PCR amplification of the V3–V4 hypervariable regions of the 16S rRNA gene. Primers 338F (5′-ACTCCTACGGGAGGCAGCAG-3′) and 806R (5′-GACTACHVGGGTWTCTAAT-3′), each containing a unique barcode, were utilized. The PCR mixture consisted of 10 μl of 2× ProTaq buffer, 2 μl of 2.5 mM dNTPs, 0.8 μl of each primer (5 μM), 0.4 μl of TransStart FastPfu DNA polymerase, 10 ng of template DNA, and sterile water to a final volume of 20 μl. The PCR protocol included initial denaturation at 95°C for 3 min, followed by 30 cycles of denaturation at 95°C for 30 s, annealing at 55°C for 30 s, and extension at 72°C for 45 s, with a final extension at 72°C for 10 min and holding at 10°C (T100 Thermal Cycler, Bio-Rad, USA). Each sample was amplified in triplicate. Post-amplification, PCR products were pooled, purified via 2% agarose gel electrophoresis, and quantified using a Synergy HTX plate reader (BioTek, USA).

For Illumina sequencing, the NEXTFLEX Rapid DNA-Seq Kit (Bioo Scientific, Austin, TX, USA) was used to prepare a library from the purified PCR products. The process involved attaching linkers to DNA fragments, purifying with magnetic beads to remove adapter self-ligation fragments, amplifying library templates via PCR, and finally recovering the PCR products using magnetic beads to obtain a purified library. Sequencing was performed on the Illumina NextSeq 2000 PE300 platform by Shanghai Meiji Biomedical Technology Co., Ltd.

To comply with sequencing requirements, samples were combined in appropriate ratios. Library preparation for PacBio sequencing used the SMRTbell Prep Kit 3.0, involving DNA damage repair, end repair, and adapter ligation. Sequencing was conducted on the PacBio Sequel IIe platform by Shanghai Meiji Biomedical Technology Co., Ltd. Raw sequencing data were stored in the NCBI Sequence Read Archive (SRA) under project PRJNA1143185 and will be publicly accessible at (https://www.ncbi.nlm.nih.gov/sra/PRJNA1143185) upon release.

#### 2.2.3 High-throughput sequencing data analysis

We used Fastp (v0.19.6) and FLASH (v1.2.11) to assess and process raw paired-end sequencing data. The steps were as follows:

(1) Quality trimming: Sequences with tail quality scores below 20 were removed, and a 50 bp sliding window was applied. If the average quality within the window fell below 20, trailing bases were trimmed. Sequences shorter than 50 bp post-trimming and those containing “N” bases were discarded.
(2) Read merging: Paired-end reads were merged into a single sequence with a minimum overlap of 10 bp.
(3) Mismatch tolerance: For merged sequences, a maximum mismatch rate of 0.2 in overlapping regions was allowed, and non-aligned sequences were discarded.
(4) Sample identification and orientation: Samples were differentiated based on barcodes and primers at the sequence ends. Barcodes were processed with zero error tolerance, while primers allowed up to two mismatches. Sequence orientation was adjusted accordingly.

UPARSE (v11) was used to process concatenated sequences, cluster them into operational taxonomic units (OTUs) at a 97% similarity threshold, and remove chimeric sequences. Taxonomic annotation was performed using the RDP classifier (v2.13) with the Silva 16S rRNA gene database (v138), with a 70% confidence threshold for species identification. OTU spikes were identified and extracted, and standard curve equations were established based on spike counts in the DNA sequencing data per sample to calculate absolute copy numbers for each taxonomic group. Finally, 16S copy number correction was applied using rrnDB.

#### 2.2.4 Statistical analysis

Post-spike DNA additive filtration, data analysis was performed using several computational methods. Alpha diversity was evaluated using the Shannon index via Mothur, and intergroup differences were analyzed using the Wilcoxon rank-sum test. For β-diversity, principal component analysis (PCA) and non-metric multidimensional scaling (NMDS) were conducted based on Curtis distance matrices. Differential bacterial taxa were identified using LEfSe with an LDA score threshold of >2 and *P* < 0.05.

In metabolomic analysis, differential metabolites were identified using orthogonal partial least squares discriminant analysis (OPLS-DA), with variable importance in projection (VIP) scores >1 and *P* < 0.05 from Student's *t*-tests. To control for multiple comparisons, the Benjamini–Hochberg false discovery rate (FDR) correction was applied, with significance defined as FDR-adjusted *q* < 0.05. Functional pathway enrichment was assessed using Fisher's exact test in Python's scipy.stats package.

### 2.3 Metabolomics detection methods

#### 2.3.1 Sample preparation

Transfer either a 50 mg solid or 100 ml liquid sample into a 1.5 ml centrifuge tube. Add 400 μl of a 1:1 (v/v) acetonitrile–methanol mixture. Mix by vortexing for 30 s, followed by ultrasonic extraction at 5°C and 40 kHz for 30 min. Incubate the mixture at −20°C for 30 min, and then centrifuge at 4°C and 13,000 × *g* for 15 min to separate the supernatant. Dry the supernatant under nitrogen gas and reconstitute the residue in 120 μl of a 1:1 (v/v) acetonitrile–water solution. Re-extract the sample by sonication at 5°C and 40 kHz for 5 min, followed by centrifugation at 4°C and 13,000 × *g* for 5 min. Transfer the final supernatant to an injection vial for analysis.

#### 2.3.2 Quality control (QC) samples

QC samples were prepared by pooling equal volumes of metabolites from each sample. During instrumental analysis, a QC sample was injected every 5–15 samples to monitor the reproducibility of the analytical process.

#### 2.3.3 LC-MS/MS analysis, substance identification, and bioinformatic analysis

Sample analysis was performed using a UHPLC-Q Exactive HF-X system (Shanghai Meiji Biomedical Technology Co., Ltd.). Raw LC-MS data were processed in Progenesis QI for baseline filtering, peak detection, integration, retention time correction, and alignment, generating a data matrix with retention time, *m*/*z* values, and peak intensities. This matrix was cross-referenced with public metabolomic databases (HMDB and METLIN) and Meiji's proprietary database for metabolite identification. Processed data were uploaded to the Meiji Cloud platform for further analysis.

Data preprocessing involved applying the 80% rule to handle missing values, ensuring at least 80% of non-zero variables were present before imputing missing values with the minimum from the original matrix. Peak intensities were normalized using sum normalization to account for variations in sample preparation and instrument performance. Variables with an RSD exceeding 30% in QC samples were log10-transformed to finalize the data matrix.

For multivariate analysis, the ropls package in R was used to perform PCA and OPLS-DA on the preprocessed data, with model stability assessed through seven rounds of cross-validation. Differential metabolites were identified based on VIP scores from the OPLS-DA model and *p*-values from Student's *t*-tests, with significance defined as VIP >1 and *p* < 0.05. Metabolic pathway enrichment was analyzed using Fisher's exact test in Python's scipy.stats package.

To investigate functional interactions between gut microbiota (GM) and metabolites, microbial–metabolite interaction networks were constructed using MetaboAnalyst 5.0 and KEGG Mapper. Key enzymes and pathways were mapped to identify potential mechanistic links between specific bacterial genera and metabolic perturbations.

## 3 Results

Among 80 screened children with allergic rhinitis (AR), 16 (20%) exhibited comorbid constipation. The clinical characteristics of the two groups (age, gender, diet, medication history, etc.) showed no significant differences (*p* > 0.05) (Table 1).

### 3.1 Analysis of GM diversity results of two groups of children

#### 3.1.1 There was significant difference in PCA between the two groups of children

**Figure 1:**
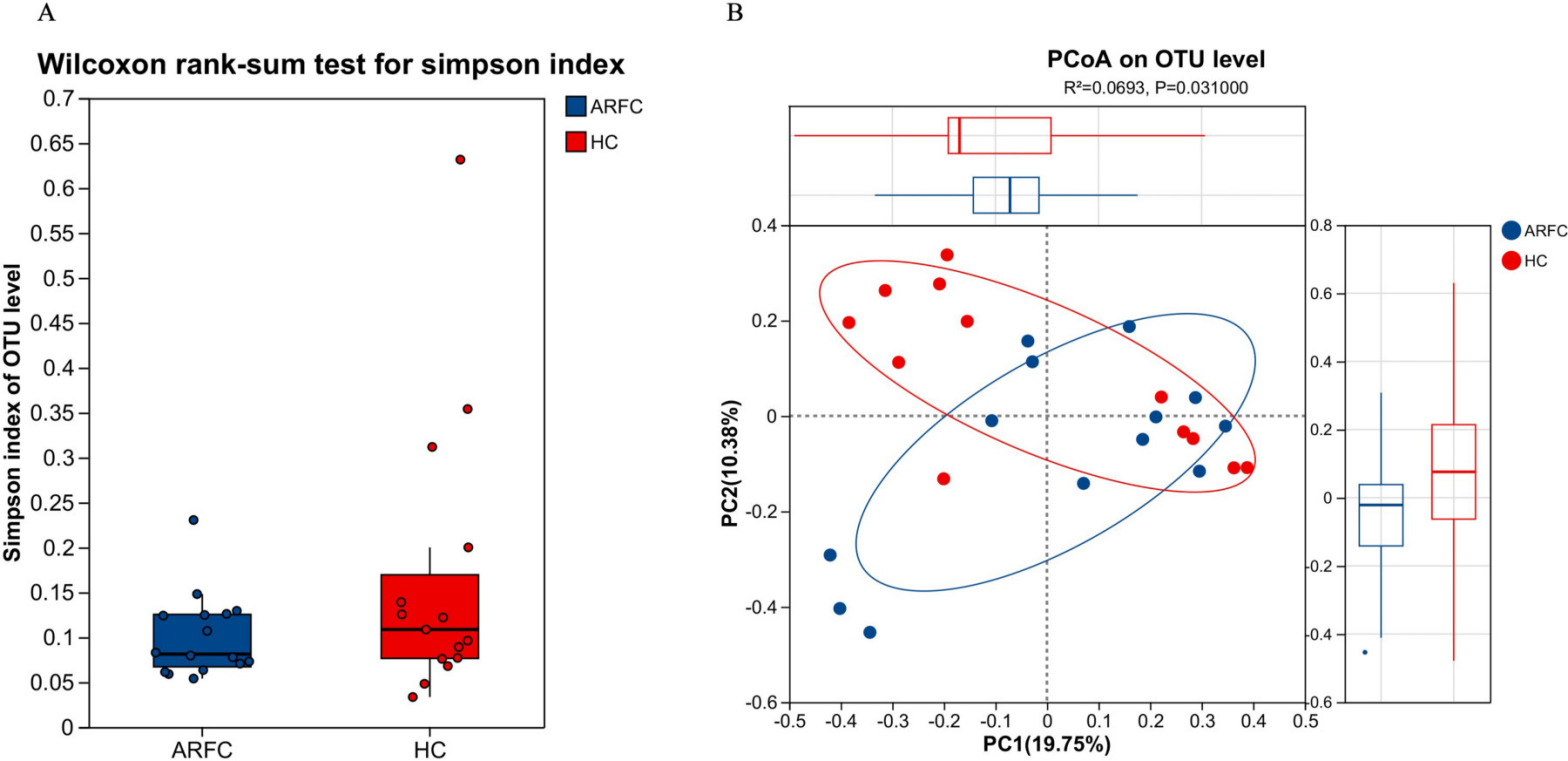
Between-group microbial diversity comparisons. Comparisons of the alpha diversity of GM in two groups. **(A)** *p*-values were calculated by using the Wilcoxon rank-sum test. **(B)** Using principal component analysis (PCA) to analyze the β-diversity of GM in 31 samples, presented by the blue circle (ARFC) and red circle (HC).

α-Diversity analysis: Using the Wilcoxon rank-sum test for the Simpson index, no significant differences in gut microbiota (GM) were observed between the two groups (*p* = 0.374) (Figure 1A); β-Diversity analysis: Principal component analysis (PCA) based on Euclidean distance matrices revealed significant differences in GM β-diversity between the two groups (*p* = 0.031) (Figure 1B).

#### 3.1.2 There were significant differences in species between the two groups of children

**Figure 2:**
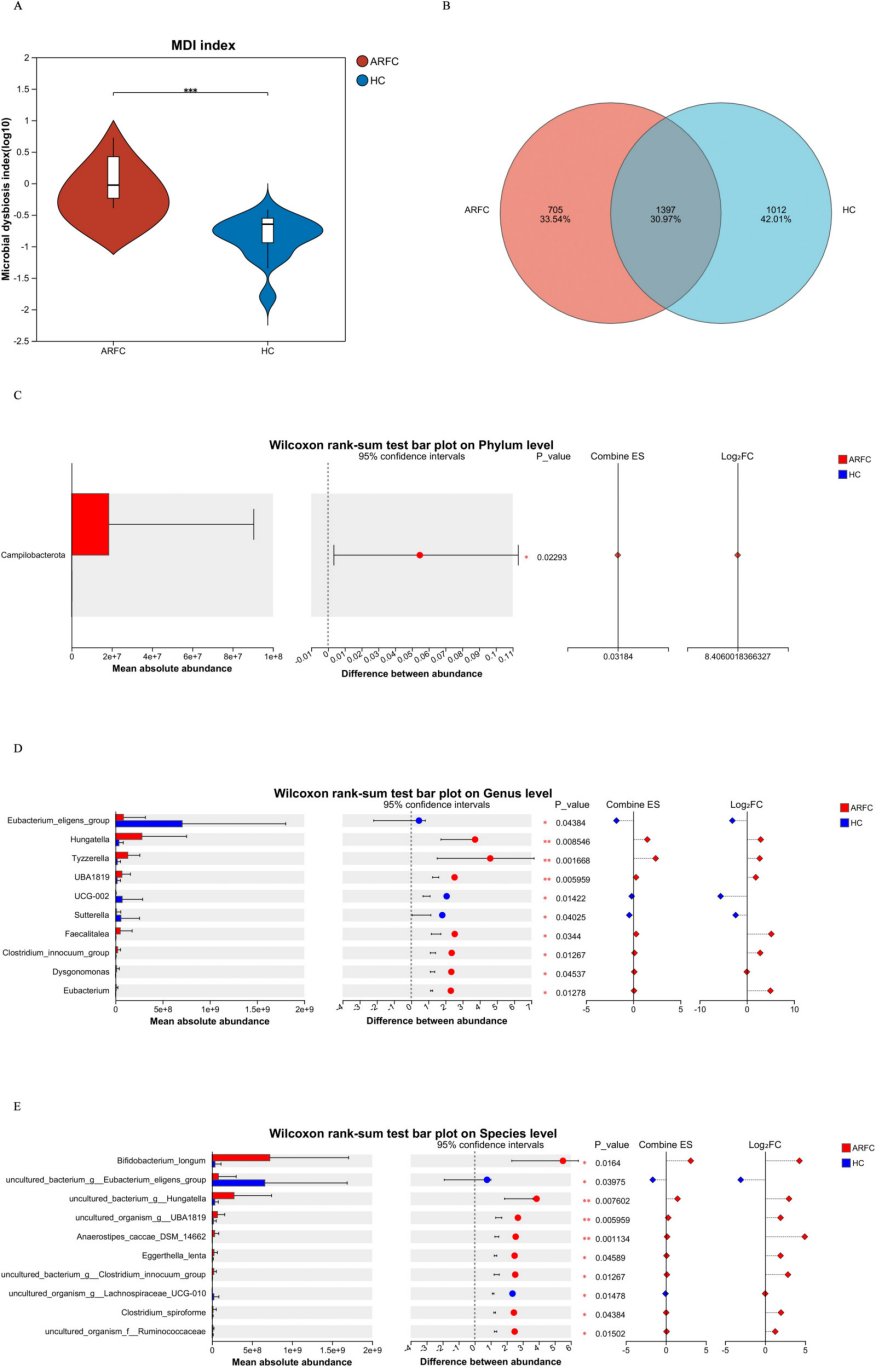
Comparison of gut microbiota composition between two groups of children. **(A)** Comparison of the gut microbiota dysbiosis index (MDI); red represents the ARFC group, and blue represents the HC group. **(B)** Comparison of the number of shared and unique OTUs in two sample groups. **(C)** Wilcoxon rank-sum test bar plot on phylum level between two groups of children; the red bar chart represents the ARFC group, and the blue bar chart represents the HC group. **(D)** Wilcoxon rank-sum test bar plot on genus level between two groups of children; the red bar chart represents the ARFC group, and the blue bar chart represents the HC group. **(E)** Wilcoxon rank-sum test bar plot on species level between two groups of children; the red bar chart represents the ARFC group, and the blue bar chart represents the HC group.

The microbial dysbiosis index (MDI) values exhibited significant differences between the two groups (*p* = 0.000) (Figure 2A). Analysis of shared and unique OTUs revealed that 1,397 OTUs were common to both groups, with 705 unique to the ARFC group and 1,012 unique to the HC group (Figure 2B).

The abundance of *Campylobacter* was significantly higher in the ARFC group compared with the HC group (*p* = 0.023) (Figure 2C). The absolute abundance of the *Eubacterium eligens* group, *UCG-002*, and *Sutterella* was lower in the ARFC group than that in the HC group (*p* < 0.05), while the absolute abundance of *Hungatella*, *Tyzzerella*, *UBA1819*, *Faecalitalea*, and the *Clostridium innocum* group was higher (*p* < 0.05) (Figure 2D). Additionally, the absolute abundance of *Bifidobacterium longum*, *DSM 14662*, and *Eggerthella lenta* was higher in the ARFC group, whereas that of the *Eubacterium eligens* group and *Lachnospiraceae UCG-010* was lower (*p* < 0.05) (Figure 2E).

#### 3.1.3 Significant correlation between intestinal flora and environmental factors

**Figure 3:**
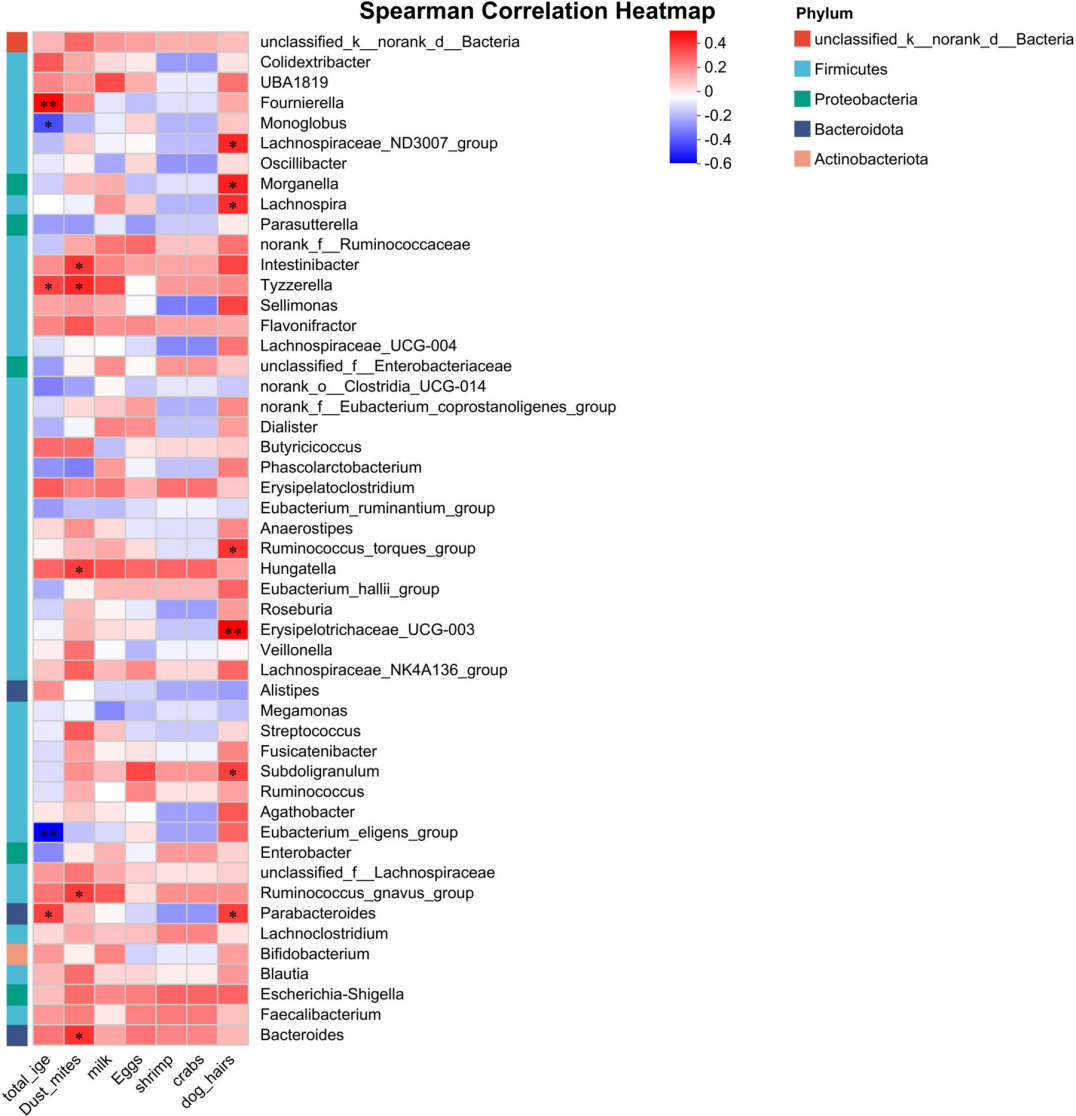
The correlation analysis between significant differences in bacterial genera and allergens in ARFC children showed that red represents a positive correlation and blue represents a negative correlation. *p < 0.05, **p < 0.01.

Correlation analysis between bacterial genera and clinical phenotypes in the ARFC group revealed the following associations (Figure 3E):

**Positive correlations** were observed between (1) dust mites and *Bacteroides*, the *Ruminococcus gnavus* group, *Hungatella*, *Tyzzerella*, and *Intestinibacter*; (2) dog hair and *Parabacteroides*, *Subdoligranulum*, *Erysipelotrichaceae* UCG-003, the *Ruminococcus torques* group, *Lachnospira*, *Morganella*, and the *Lachnospiraceae* ND3007 group; and (3) total IgE and *Fournierella*, *Tyzzerella*, and *Parabacteroides*.
**Negative correlations** were observed between total IgE and the *Eubacterium eligens* group and *Monoglobus*.

These results indicate that total IgE, dust mites, and dog hair—common allergens in ARFC children—are significantly associated with gut microbiota (GM), with most correlations being positive.

### 3.2 Analysis of untargeted metabolomics results in two groups of children

#### 3.2.1 Comparison of GM metabolite composition and differences between the two groups of children

**Figure 4:**
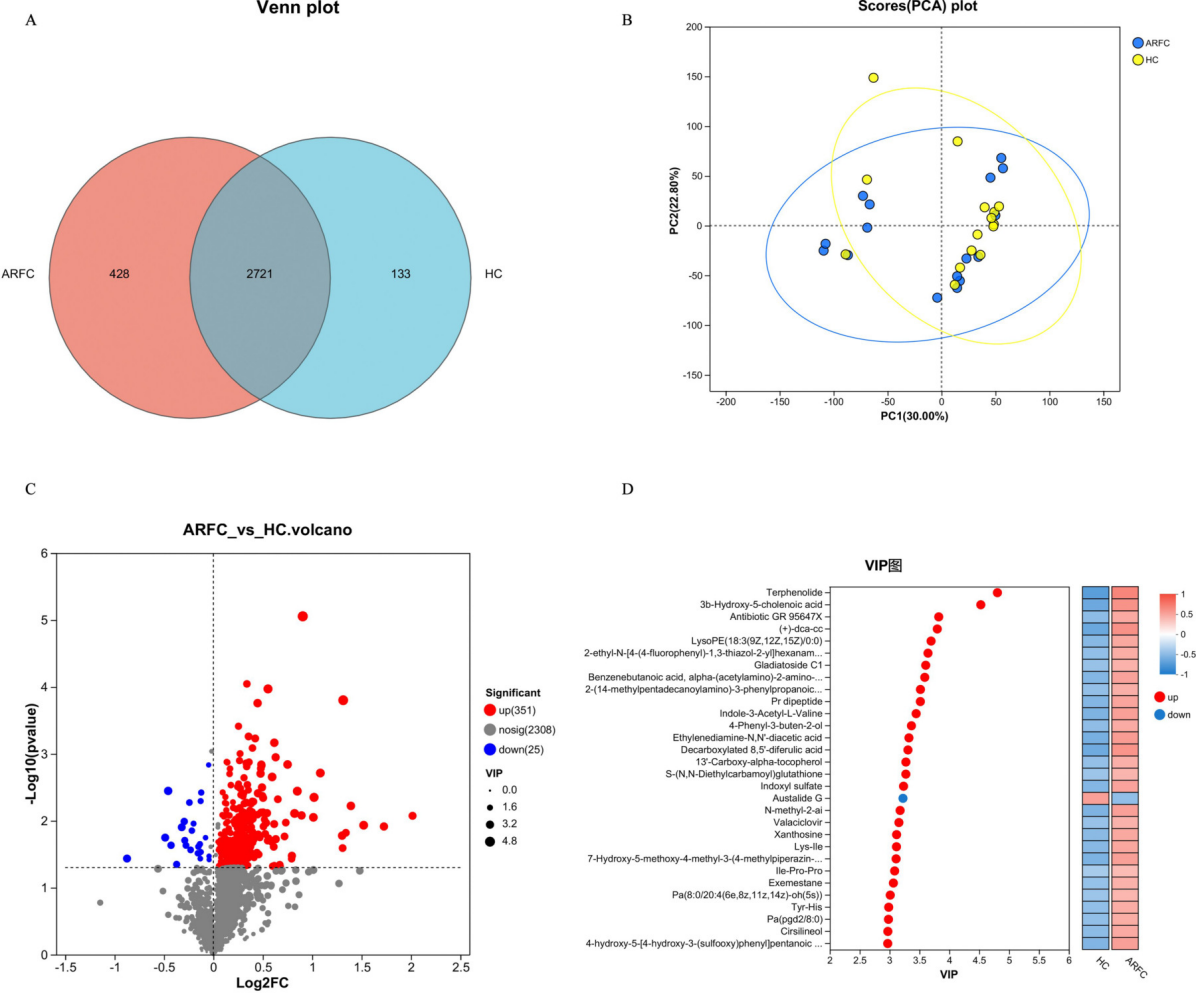
Metabolite composition and differences between the ARFC group and HC group. **(A)** A Venn diagram showing the unique and shared metabolite in two groups. **(B)** Using principal component analysis (PCA) to analyze metabolites in 31 fecal samples, presented by the blue circle (ARFC) and yellow cube (HC). **(C)** Using tools to screen for differential metabolites between two groups and draw a volcano plot, with red metabolites upregulated in the ARFC group and blue metabolites downregulated. **(D)** Using OPLS-DA/PLS-DA as a supervised model, different changes in predicted pairings were tested through sevenfold cross-validation. VIP analysis was performed using the variable projection importance of the first principal component to obtain the results of VIP metabolites.

Metabolites were extracted from 31 samples and subjected to quality control and data preprocessing. A Venn diagram illustrated that 2,721 metabolites were shared between the two groups, with 428 unique to the ARFC group and 133 unique to the HC group (Figure 4A). Principal component analysis (PCA) revealed distinct metabolic profiles between the groups, indicating significant intragroup similarity and intergroup differences (Figure 4B).

Orthogonal partial least squares discriminant analysis (OPLS-DA) was used to identify differential metabolites based on VIP values, fold changes, and *p*-values, with a volcano plot highlighting these differences (Figure 4C). A total of 351 metabolites were upregulated and 25 were downregulated in the ARFC group compared with the HC group (FDR-adjusted *q* < 0.05, VIP >1) (Supplementary Table S1). Key upregulated metabolites included aromatic amino acids (phenylalanine, tyramine, tryptamine), branched-chain amino acids (valine), short-chain fatty acids (acetic acid), neurotransmitters (epinephrine, normetanephrine), and bile acids.

OPLS-DA/PLS-DA models, validated through sevenfold cross-validation, demonstrated robust predictive capability. VIP analysis of the first principal component identified metabolites critical for classification, such as terphenolide and 3b-hydroxy-5-cholenoic acid, which were upregulated in the ARFC group. In contrast, metabolites such as Austalide G were downregulated in the ARFC group but upregulated in the HC group (Figure 4D).

#### 3.2.2 ARFC children were enriched in tryptophan metabolism pathways

**Figure 5:**
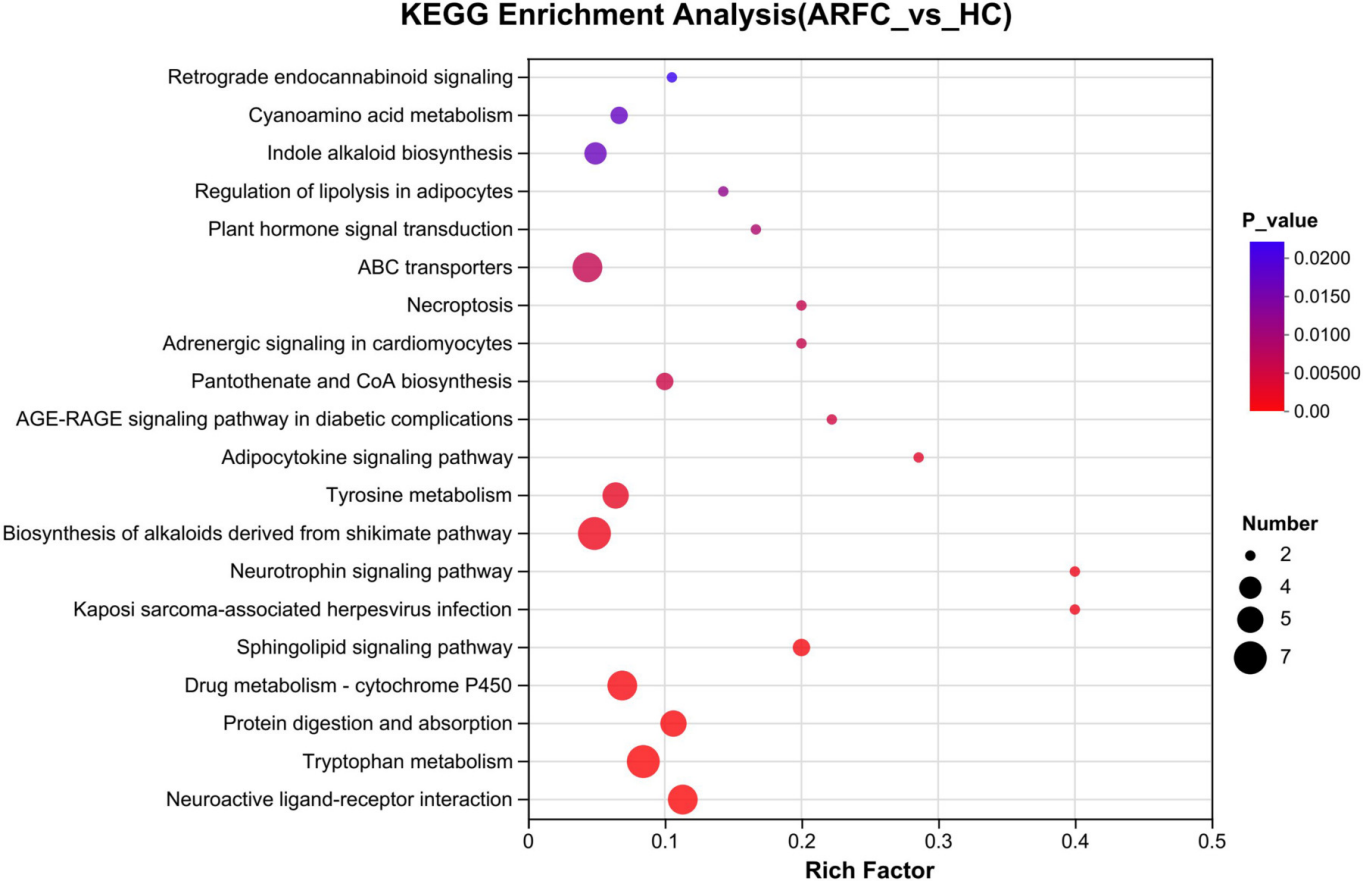
Comparison of metabolic product enrichment pathways between two groups. Comparison of the metabolites generated from 31 samples to the KEGG pathway database to determine the KEGG functional pathways involved in all metabolites.

Compared with the HC group, the ARFC group exhibited significant enrichment in metabolic pathway abnormalities, particularly in tryptophan metabolism and tyrosine metabolism. Additionally, pathways such as the biosynthesis of alkaloids derived from the shikimate pathway, neuroactive ligand–receptor interaction, ABC transporters, protein digestion and absorption, and cytochrome P450-mediated drug metabolism were also affected (Figure 5).

### 3.3 Integrated analysis of gut microbiota–metabolite interactions via metabolic modeling and bioinformatic prediction

**Figure 6:**
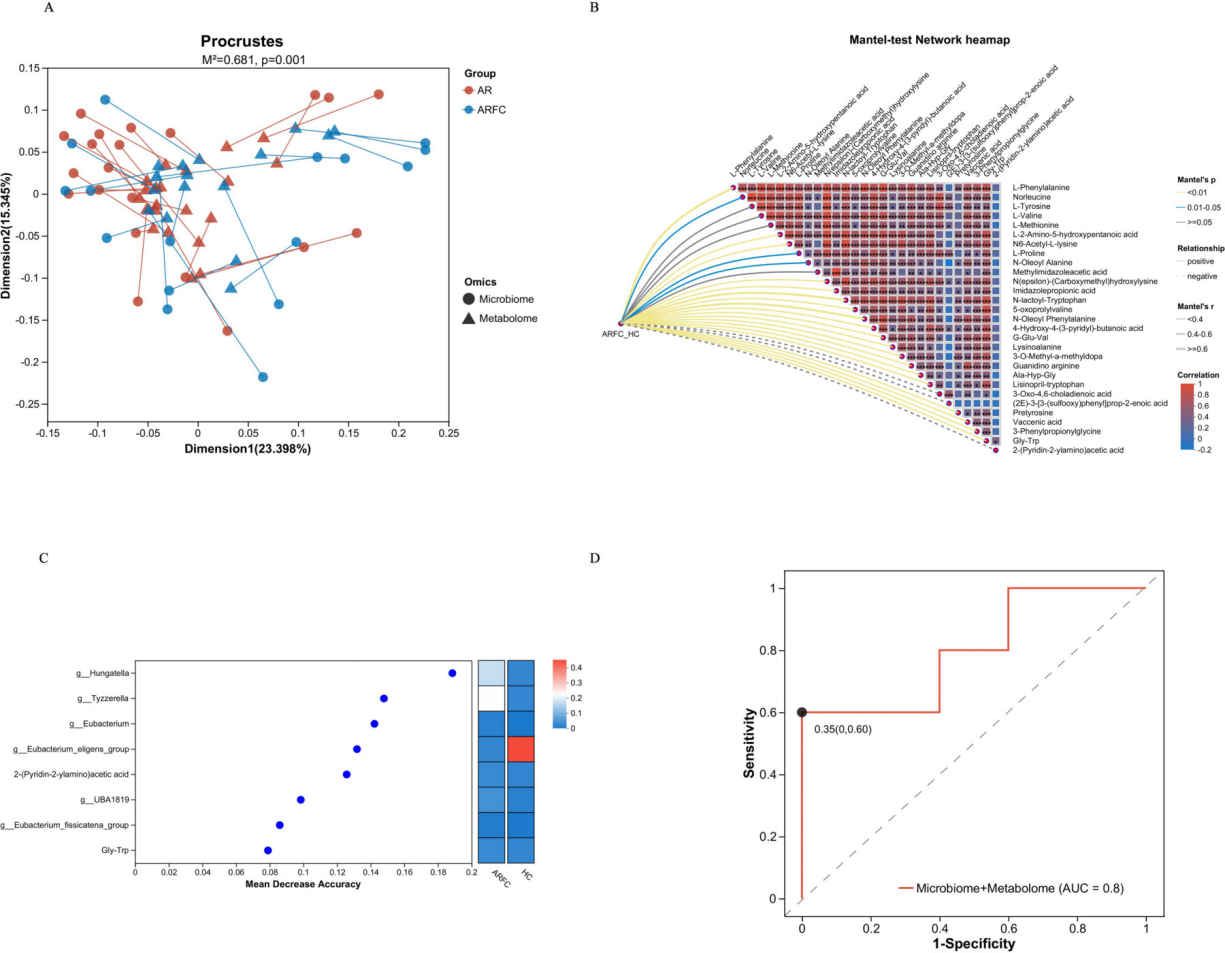
The bacterial genera with significant changes in the ARFC group children are positively correlated with the metabolism of multiple amino acids. **(A)** Procrustes analysis showed that the changes in GM and metabolites in the two groups were consistent, and there was a significant difference between the two groups (*p* = 0.001). **(B)** The Mantel test network heatmap suggested that there was a significant positive correlation between GM and metabolites in ARFC children (e.g., L-phenylalanine, N-lactoyl-tryptophan, and N-oleoyl phenylalanine). **(C)** Random forest model calculated the features to obtain the best model of GM combined metabolites (AUC = 0.8). **(D)** Random forest model confirmed that *Hungatella*, *Tyzzerella*, etc. were ARFC groups’ key GM in children.

To explore the functional interplay between gut microbiota (GM) and metabolites in children with allergic rhinitis and constipation (ARFC), we integrated metabolic pathway modeling and bioinformatic network analysis. Procrustes analysis confirmed significant concordance between GM and metabolite profiles (*p* = 0.001) (Figure 6A), indicating coordinated shifts in microbial and metabolic landscapes. Mantel tests further revealed strong positive correlations between *Hungatella*, *Tyzzerella*, and specific metabolites such as L-phenylalanine, N-lactoyl-tryptophan, and N-oleoyl phenylalanine (*r* = 0.68–0.72, *q* < 0.05) (Figure 6B).

Using genome-scale metabolic models (GEMs) and KEGG Mapper, we predicted microbial contributions to aromatic amino acid (AAA) metabolism. *Hungatella* and *Tyzzerella* were associated with key enzymes in tryptophan catabolism, such as tryptophan hydroxylase (EC:1.14.16.4) and indoleamine 2,3-dioxygenase (EC:1.13.11.52), which align with elevated levels of neuroactive tryptophan derivatives in ARFC children. *Bifidobacterium longum* was linked to phenylalanine hydroxylase (EC:1.14.16.1), supporting its role in producing phenylalanine-derived metabolites such as tyrosine and phenylethylamine.

**Figure 7:**
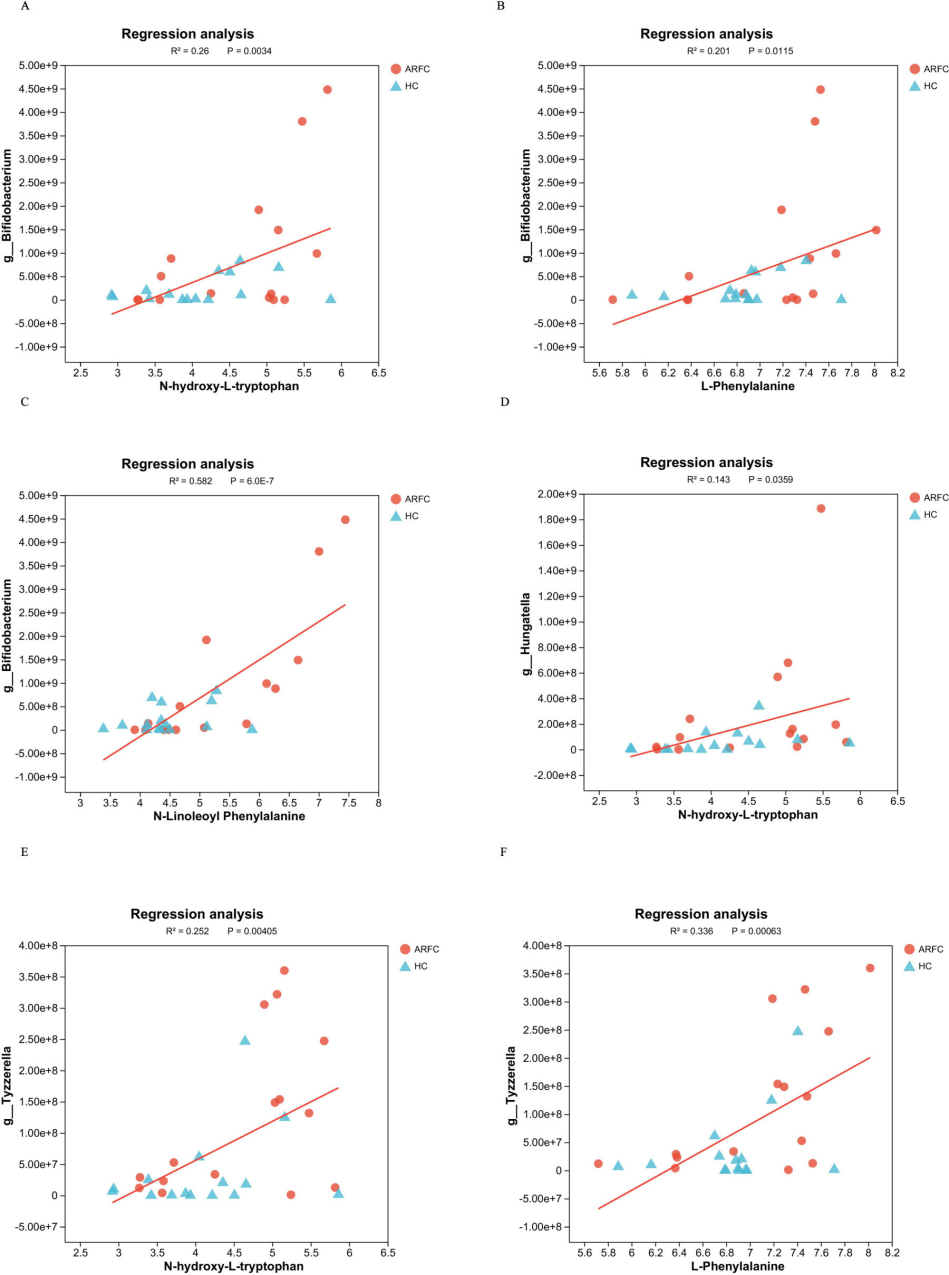
Linear regression analysis showed that there was a significant positive correlation between intestinal metabolites (including L-Phenylalanine, N-hydroxy-L-tryptophan, and N-Linoleoyl Phenylalanine) and significantly different GM (including *Bifidobacterium*, *Hungatella*, and *Tyzzerella*) in ARFC group. Regression analysis of metabolite indicators in ARFC (red circles) and HC (blue triangles) groups. (**A**–**F**) Subplots represent relationships between different metabolite pairs (e.g., N-hydroxy-L-tryptophan). Axes labels indicate metabolite concentrations (× 10^9^). Regression statistics (R^2^, R, and P-values) are displayed within each subplot.

Network analysis via MetaboAnalyst 5.0 showed that *Hungatella* and *Tyzzerella* co-occurred with metabolites involved in tryptophan/tyrosine pathways (Figures 7A–F). For example, *Hungatella* exhibited strong connectivity to N-lactoyl-tryptophan (*q* = 0.018), an anti-inflammatory precursor, while *Tyzzerella* correlated with pro-inflammatory kynurenine (*q* = 0.012), consistent with its potential role in Th17 activation.

A random forest model combining key genera (*Hungatella*, *Tyzzerella*, *Bifidobacterium*) and metabolites (L-phenylalanine, N-oleoyl phenylalanine) demonstrated high diagnostic accuracy (AUC = 0.8) (Figure 6C). Recursive feature elimination with cross-validation (RFECV) identified these taxa as central discriminators of ARFC (Figure 6D), underscoring their mechanistic importance.

These findings demonstrate that GM dysbiosis in ARFC children drives AAA metabolism through specific enzyme interactions, promoting the production of neuroactive and pro-inflammatory metabolites. This integrative approach bridges correlation and causality, offering a framework for targeting GM–metabolite axes in therapeutic strategies.

## 4 Discussion

### 4.1 Research Status

Mothers with constipation have a significantly higher risk of having offspring with AR (6), and individuals with constipation are twice as likely to develop AR compared with those without constipation (7). Constipation may influence immunity by altering gut microbiota (GM) and intestinal permeability, leading to excessive secretion of pro-inflammatory biomarkers such as chemokines and cytokines (22, 23), thereby inducing or exacerbating allergic symptoms. In our study cohort, 20% of AR children (16 out of 80 screened) presented with comorbid constipation. Both AR and constipation are associated with GM dysbiosis (9–13, 24), which may represent a common pathological mechanism. Most studies (9, 12, 19) have reported a significant reduction in the abundance of beneficial bacteria, such as *Bifidobacteria* and *Lactobacilli*, in the intestines of children with AR and constipation. Metabolomic studies have also revealed abnormal metabolism of amino acids (e.g., tryptophan and histidine) and lipids (e.g., arachidonic acid) in the AR population. Furthermore, changes in amino acid and lipid metabolism have been observed following immunotherapy for AR symptom control (16, 17). Similarly, constipation is primarily caused by abnormalities in lipid metabolism (e.g., bile acids and related metabolites), and amino acid metabolism can also be affected (18–20).

### 4.2 ARFC children’s Gm is closely related to constipation and allergies

In the intestines of ARFC children, the absolute counts of certain bacterial genera, such as *Hungatella*, *Tyzzerella*, *UBA1819*, *Faecalitalea*, *Clostridium innocuum*, and *Bifidobacterium longum*, as well as strains such as *DSM 14662* and *Eggerthella*, were significantly increased. Previous studies (25, 26) have linked *Hungatella* and *Tyzzerella* to constipation, with the latter's abundance decreasing following probiotic intervention and constipation relief. *UBA1819*, potentially an anti-inflammatory probiotic (27), aids in regulating host immune responses and promoting intestinal health (28). *Faecalitalea* and *Clostridium innocuum* are considered potential gut pathogens (29, 30), although their exact mechanisms remain unclear. *Bifidobacterium longum* has been shown to alleviate allergic symptoms but may also contribute to constipation (31–33). *DSM 14662*, a strain of *Anaerostipes caccae*, promotes defecation and allergy prevention when its levels are elevated (34, 35). *Eggerthella lenta* can induce intestinal inflammation and allergies by activating Rorc and Th17-related genes and is involved in bile acid metabolism (36). Correlation analysis also indicated a significant link between these bacterial genera and allergens such as dust mites in ARFC children, further highlighting the close relationship between gut microbiota and allergies.

### 4.3 Unique metabolomic alterations in ARFC children

Our multi-omics approach identified *Hungatella* and *Tyzzerella* as key drivers of tryptophan metabolism dysregulation in ARFC children. Network modeling highlighted their functional links to enzymes critical for tryptophan catabolism, such as tryptophan hydroxylase, which aligns with elevated fecal levels of serotonin and kynurenine. These findings are consistent with prior studies associating *Hungatella* with neuroactive metabolite synthesis and *Tyzzerella* with intestinal inflammation via Th17 activation (37, 38).

The interaction between *Bifidobacterium longum* and phenylalanine hydroxylase supports the hypothesis that gut microbiota (GM)-driven aromatic amino acid (AAA) metabolism exacerbates both allergic and gastrointestinal symptoms. Phenylalanine derivatives may increase mucosal permeability and IgE production (15), while excessive serotonin could disrupt gut motility, perpetuating constipation (39). These mechanisms align with the dual role of AAAs in immune modulation and neural signaling (40, 41).

Metabolic modeling further validated these correlations by linking functional pathways, such as shikimate and ABC transporters, to specific microbial taxa. For example, *UBA1819*'s association with bile acid transporters (KEGG map04976) may explain elevated bile acid levels in ARFC children, which could intensify allergic inflammation via FXR/TGR5 signaling (36). Similarly, *Faecalitalea*'s role in acetate synthesis aligns with its potential to modulate T-regulatory cells (42).

In conclusion, computational modeling not only supports the observed correlations but also provides a mechanistic framework for future interventions targeting GM–metabolite axes in ARFC.

## 5 Conclusion and shortcomings

Our multi-omics analysis reveals that children with ARFC harbor a unique gut microbial signature characterized by increased *Hungatella*, *Tyzzerella*, and *Bifidobacterium longum*, alongside dysregulated aromatic amino acid metabolism. Bioinformatic modeling demonstrated that *Hungatella* and *Tyzzerella* drive tryptophan catabolism via key enzymes (tryptophan hydroxylase and indoleamine 2,3-dioxygenase), promoting neuroactive metabolites such as serotonin and pro-inflammatory kynurenine. *Bifidobacterium longum* further exacerbated phenylalanine-derived metabolite levels, potentially contributing to mucosal permeability and IgE-mediated allergic responses. The integration of GM and metabolomic data into a predictive model (AUC = 0.8) highlights the diagnostic potential of microbial–metabolite axes. However, a key limitation is the lack of comparison with AR-only or constipation-only groups. Future research should include these subgroups to disentangle the specific contributions of AR and constipation to GM dysbiosis. Future studies also should validate these mechanisms in larger populations and explore interventions targeting GM composition to alleviate dual symptoms of allergy and gastrointestinal dysfunction.

## Data Availability

The datasets presented in this study can be found in online repositories. The names of the repository/repositories and accession number(s) can be found in the article/Supplementary Material.
