# Correction: Multi-omics technology reveals the changes in gut microbiota to stimulate aromatic amino acid metabolism in children with allergic rhinitis and constipation

**DOI:** 10.3389/falgy.2025.1655751

**Published:** 2025-07-14

**Authors:** Chunyan Wang, Haiying Liu, Xiaoli Li, Wei Kong, Hui Wu, Congfu Huang

**Affiliations:** ^1^ Department of Pediatrics, The Fourth People’s Hospital of Shenzhen, Shenzhen, China; ^2^ Department of Pediatrics, Affiliated Shenzhen Maternity and Child Healthcare Hospital, Southern Medical University, Shenzhen, China; ^3^ Department of Pediatrics, Longgang District Maternity & Child Healthcare Hospital of Shenzhen City (Longgang Maternity and Child Institute of Shantou University Medical College), Shenzhen, China; ^4^ Child Healthcare Department, Panyu Maternal and Child Care Service Centre, Guangzhou, China

**Keywords:** allergic rhinitis, constipation, gut microbiota, aromatic amino acids, high-throughput absolute quantification, metabolomics

A Correction on Multi-omics technology reveals the changes in gut microbiota to stimulate aromatic amino acid metabolism in children with allergic rhinitis and constipation By Wang C, Liu H, Li X, Kong W, Wu H and Huang C (2025). Front Allergy. 6:1562832. doi: 10.3389/falgy.2025.1562832

Author Haiying Liu was erroneously assigned to affiliation “^1^Department of Pediatrics, The Fourth People’s Hospital of Shenzhen, Shenzhen, China”. This affiliation has now been removed for author Haiying Liu. The correct author affiliation order now reads as “Chunyan Wang^1†^, Haiying Liu^2†^, Xiaoli Li^3^, Wei Kong^3^, Hui Wu^4^ and Congfu Huang^3^*”.

The original version of this article has been updated.

